# Repurposed Drugs to Enhance the Therapeutic Potential of Oligodendrocyte Precursor Cells Derived from Adult Rat Adipose Tissue

**DOI:** 10.3390/cells14070533

**Published:** 2025-04-02

**Authors:** J. Pascual-Guerra, M. Torres-Rico, B. Marín-Rodríguez, M. S. Arasmou-Idrovo, A. G. García, J. A. Rodríguez-Navarro, C. L. Paíno

**Affiliations:** 1 Servicio de Neurobiología-Investigación, IRYCIS, Hospital Universitario Ramón y Cajal, 28034 Madrid, Spaincarlos_paino@yahoo.com (C.L.P.); 2 Fundación Teófilo Hernando, 28290 Madrid, Spain; 3 Departamento de Farmacología, Facultad de Medicina, Universidad Autónoma de Madrid, 28023 Madrid, Spain; 4 Departamento de Bioquímica y Biología Molecular, Facultad de Farmacia, Instituto Universitario de Investigación Neuroquímica (IUIN), Universidad Complutense de Madrid, 28040 Madrid, Spain

**Keywords:** drug repurposing, oligodendrocytes precursor cells, myelin, lentiviral transduction

## Abstract

Failure in the proliferation, recruitment, mobilization, and/or differentiation of oligodendrocyte precursor cells (OPCs) impedes remyelination in central nervous system (CNS) demyelinating diseases. Our group has recently achieved the generation of functional oligodendroglia through direct lineage conversion by expressing *Sox10*, *Olig2*, and *Zfp536* genes in adult rat adipose tissue-derived stromal cells. The present study aimed to determine whether various repurposed drugs or molecules could enhance the myelinating capacities of these induced OPCs (iOPCs). We report that kainate, benztropine, miconazole, clobetasol, and baclofen promote in vitro iOPCs migration, differentiation, and ensheathing abilities through mechanisms similar to those observed in rat neural stem cell-derived OPCs. This research supports the potential use of iOPCs as they provide an alternative and reliable cell source for testing the effects of in vitro promyelinating repurposed drugs and for assessing the molecular and cellular mechanisms involved in therapeutic strategies for demyelinating diseases.

## 1. Introduction

Along with astrocytes, oligodendrocytes (OLs) are a major component of the glial cells in the central nervous system (CNS), which for decades were considered mere silent partners to neurons. However, this view has been dramatically revised over the last two to three decades, as the importance of glial cells has been recognized due to their roles in CNS architecture, brain metabolism, and the development and maintenance of myelin sheaths, among other functions [1]. Considering this, it is unsurprising that glial impairments are implicated in the mechanisms underlying a wide range of CNS clinical conditions, including Alzheimer’s disease, Parkinson’s disease, epilepsy, schizophrenia, and demyelinating diseases [2].

In demyelinating diseases, such as multiple sclerosis (MS), there is a degeneration of OLs that wrap long-distance projecting axons in CNS, which then lose their myelin sheaths required for the fast and efficient transmission of action potentials. Oligodendrocyte precursor cells (OPCs) are the primary source of myelinating cells in the developing CNS and are recruited at demyelinated areas in adult CNS to initiate myelin reparative processes. After migrating to damaged areas, they have the potential to differentiate into mature OLs and, to a certain extent, remyelinate denuded axons. The pathophysiology of MS is primarily characterized by disruptions in the orchestrated processes of specification, proliferation, recruitment, migration, and differentiation of OPCs into mature OLs, eventually leading to the interruption of saltatory conduction of nerve impulses, which may cause cognitive, sensory, and/or motor impairments [3].

Despite the aforementioned, the vast majority of current therapeutic strategies for demyelinating diseases focus on treating the inflammatory response and the acute symptoms of refractory spasticity, fatigue, and pain [4]. As of now, there are no clinically available treatments for MS involving OLs. In response to this, numerous research groups are actively working to develop cell therapies. These therapies are based on various methods to culture expandable and functional OLs, such as reprogramming cells from induced pluripotent stem cells (iPSCs) or expressing transcription factors in embryonic fibroblasts [5,6,7]. However, the use of iPSCs may present challenges, as residual pluripotent cells could continue to proliferate and potentially form teratomas. Additionally, there are logistical and ethical considerations regarding the use of embryonic cells.

For these reasons, alternative cell sources are being sought. Somatic cells, particularly adult adipose tissue-derived stromal cells, are promising candidates for developing similar strategies. They have the advantages of rapid growth in culture and easy accessibility. Additionally, their immunomodulatory, anti-inflammatory, neuroprotective, and trophic properties, along with their potential for selective autophagy, synaptogenesis, and gliogenesis, make them potentially ideal for treating neurovascular and demyelinating diseases [8].

In this context, our research group has recently demonstrated the conversion of adult rat adipose tissue-derived stromal cells into induced oligodendroglia (as well as other phenotypes) by expressing the transcription factors Sox10, Olig2, and Zfp536 via lentiviral transduction [9].

Beyond these cell-based strategies, there is currently growing interest in drug repurposing or repositioning. This approach can be described as a set of tools, knowledge, and techniques aimed at finding new therapeutic uses for drugs that are already approved for different, often unrelated, pathologies. Given the crucial role of OPCs in demyelinating diseases, any repurposed drug identified as a therapeutic candidate should support remyelination by promoting the proliferation, migration, and/or differentiation of these cells.

Cells of the oligodendrocyte lineage express a wide variety of neurotransmitter receptors, including glutamate, γ-aminobutyric acid (GABA), acetylcholine, dopamine or cannabinoids, as well as the superfamily of nuclear receptors (e.g., steroid hormones, vitamin D3, thyroid hormone, retinoic acid, fatty acid amides, etc.), and respond to their ligands. As promising strategies in demyelinating diseases, it is worth mentioning the agonism of GABA_A_ [10] and GABA_B_ [11], the activation of metabotropic glutamate receptors (mGluRs) [12,13], the antagonism of muscarinic acetylcholine receptors (mAchRs) [14], or the activation of glucocorticoid receptors [15].

A leading group, headed by researchers Mei and Chan, developed a platform called BIMA (Binary Indicant for Myelination using Micropillar Arrays) to study the myelination-promoting activity of a wide collection of drugs. Among their notable findings, this group identified two mAChR antagonists, benztropine and clemastine, as effective drugs that enhance myelination [16]. Benztropine belongs to the group of synthetic drugs with activity as a centrally acting muscarinic antagonist, antihistamine, and dopamine reuptake inhibitor, and in clinical practice is primarily used to treat the symptoms of Parkinson’s disease [17]. However, it has recently been proposed as a drug with potential therapeutic use in demyelinating pathologies such as multiple sclerosis because it has been shown to promote the differentiation and remyelination in both OPC cultures derived from rat optic nerve and in animal models of experimental autoimmune encephalomyelitis or cuprizone demyelination [18].

Mei and colleagues paved the way for other research groups that employed similar strategies and reported ‘new uses for old drugs’, such as clobetasol (glucocorticoid receptors agonist) [19], miconazole (MAPK route activator) or taurine [20,21], for the hypothetical treatment of demyelinating diseases. Miconazole is a synthetic antifungal imidazole that is used clinically to treat superficial fungal infections effectively and safely [22]. Recent studies suggest that miconazole also promotes OPC migration, differentiation, remyelination, axonal regeneration, and/or neuroprotection after CNS [23] and PNS damage [24,25].

Other research groups have also identified various promising compounds through literature review or database mining, based on their therapeutic potential. These compounds are significant regulators of OPC differentiation into mature oligodendrocytes and axonal myelination. Consequently, both the GABA_B receptor agonist baclofen [26] and the AMPA receptor agonist kainate [27] were posited as viable candidates for the treatment of demyelinating diseases. While kainate is not traditionally classified as a repurposed drug, this study has opted to include it because of our previous studies [27]. Furthermore, it is mainly recognized as an excitotoxic drug and neurotoxin, for which there are no studies on its possible therapeutic activity nor pharmacodynamics. However, we have decided to include kainate in the present study because its activity is equivalent to the other, repurposed, drugs tested in this work.

Here we demonstrate that the repurposed drugs/molecules benztropine, miconazole, clobetasol, baclofen, and kainate promote the in vitro migration and differentiation of oligodendrocyte precursor cells derived from adult rat adipose tissue. We further demonstrate that these compounds also enhance the in vitro myelination capabilities when co-cultured with rat dorsal root ganglion neurons.

## 2. Materials and Methods

A list of commercial product references is provided in Supplementary Table S1.

### 2.1. Animals and Procedures

All procedures involving animals were carried out by qualified personnel in accordance with Directive 2010/63/EU of the European Union on the protection of animals used for scientific purposes, as well as its transposition into Spanish law (RD53/2013). Rats were bred at the animal facilities of Hospital Universitario Ramón y Cajal (ES280790002001). For tissue collection, the rats were first deeply anesthetized with isoflurane and then euthanized by decapitation. Approval from the ethics committee was not required for these procedures.

### 2.2. Oligodendrocyte Precursor Cells Induction

Induced oligodendrocyte precursor cells were generated by expressing the transcription factors Tet-O-FUW-Sox10 (Addgene plasmid #45843), Tet-O-FUW-Zfp536 (Addgene plasmid #45845), and Tet-O-FUW-Olig2 (Addgene plasmid #30131) via lentiviral transduction in adult rat adipose tissue-derived stromal cells (ADSCs). For a detailed protocol, please refer to [9].

Briefly, adipose-derived stromal cells (ADSCs) were cultured from the inguinal fat pads of Sprague–Dawley rats weighing 220–250 g. The tissue was disaggregated through mechanical and enzymatic means, and the cells were cultured in Minimal Essential Medium, alpha modification (Sigma-Aldrich, Madrid, Spain) supplemented with 20% FBS, glutamine, and antibiotic/antimycotic (all from Gibco, Madrid, Spain) and non-essential amino acids (Sigma-Aldrich), hereafter referred to as α20 medium. These cells proliferated and reached near-confluence after 4–5 days, at which point they were frozen in aliquots in FBS with 10% DMSO.

Self-inactivating, replication-incompetent lentiviruses were produced in HEK293T cells by co-transfecting the lentiviral plasmid, the packaging plasmid psPAX2, and the envelope vector pCMV-VSV-G using Lipofectamine 2000 (ThermoFisher, Madrid, Spain). The viruses were titrated using the Lentivirus qPCR kit (Applied Biological Materials, Madrid, Spain), which contains reverse transcriptase, standards, and primers specific for the lentiviral 5′-LTR, employing the LightCycler 480 (Roche Diagnostics, Rotkreuz, Switzerland) with the SybrGreen I Master kit (Roche Applied Science, Madrid, Spain). For lentiviral transduction, an aliquot was thaw and ADSC were seeded at 15,000 cells/cm^2^ in 24-well plates and allowed to grow for 24 h in α20 medium. Then, ADSC underwent lentiviral transduction with tetracycline-inducible *Sox10*, *Olig2*, and *Zfp536* (SOZ) genes.

### 2.3. iOPCs Culture

To induce transgene expression, SOZ-transduced ADSCs (ADSC-SOZ) were cultured in Neurobasal medium supplemented with B27 (Invitrogen, Madrid, Spain, here referred to as NBB27), doxycycline hydrochloride (1 μg/mL, Fisher Scientific, Madrid, Spain), EGF (20 ng/mL), bFGF (20 ng/mL), PDGF-AA (10 ng/mL, all three from Peprotech EC, ThermoFisher, Madrid, Spain), and d-biotin (10 ng/mL, Sigma-Aldrich). Various assays, including immunocytochemistry and RT-qPCR, were conducted over time to characterize the culture [9].

This procedure was continued until nearly all cells showed small, spheric bodies with few short branches, exhibited intense nuclear Sox10 staining, and more than 50% of cells tested positive for the O4 marker. Cultures at this stage—requiring approximately 100 days of transgene induction—doubled the cell population every 2 days and were considered to contain iOPCs.

### 2.4. NSC Culture

Neural stem cell-derived OPCs (NSC-OPCs) served as the positive control in most of the experiments, which were carried out in parallel with iOPCs throughout the study. These cells were obtained from neonatal Sprague–Dawley rats (P3–P6). Regarding the NSCs, several batches were conducted throughout the project; however, only the last five batches have been included in this work. Accordingly, cerebral cortices from 3–6-day-old postnatal rat pups were used—one per culture, obtained from different pregnant rats—allowing us to use five rat pups without having to sacrifice the mothers.

The cerebral hemispheres were separated by cutting the skull through the sagittal midline, and the meninges were carefully removed. Once the cortex was exposed, a portion of approximately 0.5 cm^2^ from the temporoparietal area was dissected out, incubated for 10 min at RT in Accutase (1:4 in Hank’s balanced salt solution) and smoothly triturated using sterile filter tips attached to a P1000 automatic pipette (Labclinics, Madrid, Spain). Dispersed tissue was passed through a 100 μm nylon mesh cell strainer (BD Biosciences, Franklin Lake, NJ, USA) into a 50 mL conical tube (Falcon, Thermo Fisher Scientific, Madrid, Spain) and centrifuged for 3 min at 400× *g*. The supernatant was discarded, and the pellet was resuspended in NBB27 medium supplemented with EGF (20 ng/mL) and FGFb (20 ng/mL). Finally, cells were cultured as floating aggregates in flasks (Nunc, Thermo Fisher Scientific).

This culture was maintained for at least one week, with culture medium changes every 2–3 days. During these changes, the floating cells were collected, centrifuged, and reseeded in a different flask containing NBB27 medium supplemented with EGF (20 ng/mL) and bFGF (20 ng/mL). At this stage, the aggregated NSCs (neurospheres) were dispersed by passing them through a P1000 automatic pipette fitted with a fire-polished, sterile filter tip. By day 10, the medium was supplemented with PDGF-AA (10 ng/mL), which promoted the generation of oligospheres (OPCs-enriched cellular aggregates).

For the pharmacological experiments, the oligospheres were digested with accutase and disaggregated mechanically as previously described. They were then maintained in NBB27 medium supplemented with either EGF + bFGF + PDGF-AA for NSC-OPCs (O4^+^ enriched culture) or triiodothyronine (T_3_, Sigma-Aldrich) for differentiated oligodendroglia (O1^+^ enriched culture).

### 2.5. Cell Migration Assay

Cell migration was assessed by an adaptation of the method developed by Frost, Milner, and ffrench-Constant to demonstrate the in vitro proliferative capacity of OPCs [28]. On the day before an assay, the surfaces of P24 wells were coated by incubating for 1 h at 37 °C with 300 µL of Geltrex matrix (Gibco) diluted 1:100 in sterile cold water; after draining the liquid, the plates were allowed to dry out in a laminar flow hood.

**Figure 1:**
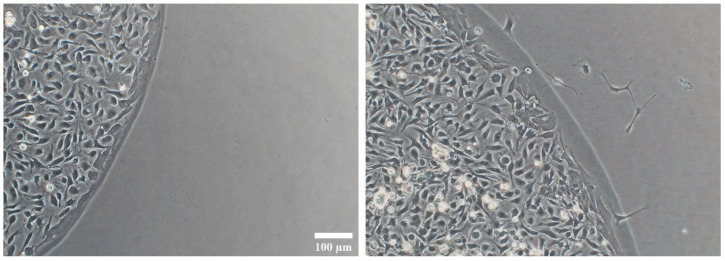
Seeding in agarose drops and migration on Geltrex matrix. At 24 h post-seeding, the cells were settled on the matrix and confined to the agarose drop boundaries (**left**). By 48 h, some cells had emerged from the agarose drop and migrated in all directions of the matrix surface (**right**). Magnification: 10×. Scale bars: 100 µm.

For seeding, 750,000 cells were resuspended in 30 µL of NBB27 plus 15 µL of 1% sterile low-melting agarose (Fisher Bioreagents, Madrid, Spain) solution, and 1.5 µL of this cell suspension was seeded as a semi-spherical drop at the centre of Geltrex pre-coated wells (25,000 cells/drop). The seeding was performed in the 12 inner wells of pre-warmed plates placed on top of a thermal plate at 39 °C (JP Selecta, Barcelona, Spain) inside a horizontal laminar flow hood; in addition, the 12 peripheral wells of each plate were filled with sterile water in order to prevent cell drops from drying out. Plates were then taken to incubators inside humidity chambers and cells were allowed to settle and attach to the surface of Geltrex matrix for 15 min. Then, the chambers with plates were taken to a 4 °C fridge for additional 15 min so that agarose drops solidified with the cells confined inside. Thereupon, 50 µL of NBB27 + EGF (20 ng/mL) + FGFb (20 ng/mL) + PDGF-AA (10 ng/mL) + biotin (10 ng/mL) + doxycycline (1 mg/mL) was carefully added around the drops, in the case of the iOPCs, or the same medium without doxycycline in the case of the OPCs. The plates were returned to the incubator for 2–3 h. Finally, the medium was completed up to 400 µL with the same cocktail. The first cells were observed crossing the boundary of the agarose drop after 24 h, migrating in all directions across the Geltrex-coated well surface thereupon. Then, pharmacological treatments were applied (Figure 1).

During the one-week experiment period, the culture medium and the corresponding pharmacological treatment were refreshed every three days. The schedule depended on the day of the week the experiment began. For example, the droplet culture was typically initiated on a Monday. On Tuesday, the medium was replaced with fresh medium containing the drug. The next medium change occurred on Friday, allowing migration analysis to take place on the following Monday. Therefore, the pharmacological treatment was applied at two distinct time points during the experiment. After 7 days of this treatment, the migratory cells were counted. To do this, the cells were fixed in 4% paraformaldehyde, O4 immunolabeling was performed, and cell nuclei were counterstained with 10^−5^ M bisbenzimide-Höechst 33342 (Sigma-Aldrich). Migratory cells were counted in eight directions around the drop using a 10× objective on an inverted fluorescence microscope (Telaval 3 (Carl Zeiss, Jena, Germany)).

**Figure 2:**
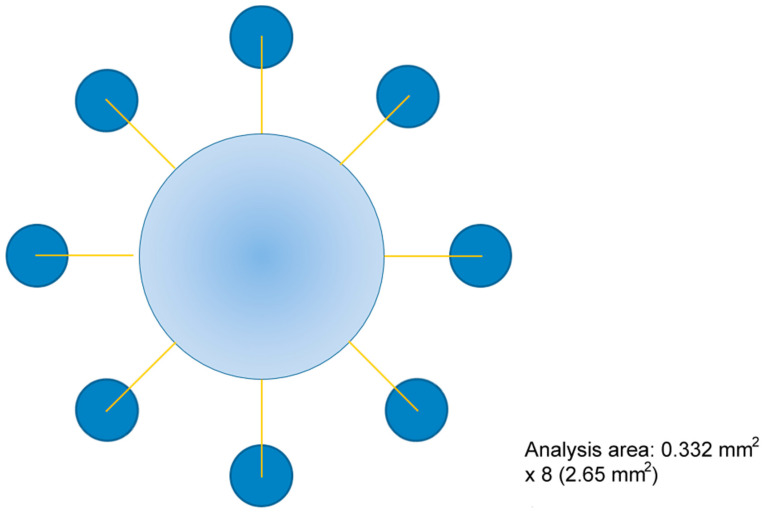
Scheme for counting migrating cells. After a week of treatment, immunocytochemistry (ICC) was conducted to count nuclei and O4^+^ cells in eight directions radiating from the agarose drop (light blue large circle) at a distance of 1100 µm from the agarose border. Analysis area (dark blue circles): 0.332 mm^2^ × 8 (2.65 mm^2^). This was followed by calculating the average number of migrating cells inside sampled areas for each pharmacological treatment.

Bisbenzimide-stained nuclei and O4^+^ cells were counted within circular ROIs (regions of interest) measuring 0.332 mm^2^. The distance from the drop’s edge to the centre of these circular ROI was 1100 µm, and the ROI’s radius was 325 µm. Cells within these ROI were considered migratory, and their number provided an estimate of their migratory capacity (Figure 2).

### 2.6. Immunocytochemistry

Commonly, the cells were seeded on sterile 12 mm circular glass coverslips placed inside 24-well plates at a density of 44 cells/mm^2^ (approximately 5000 cells per coverslip) in the case of iOPCs and OPCs or 177 cells/mm^2^ for co-culture with DRG neurons (approximately 20,000 cells per coverslip). For fractal analysis of morphological complexity (see below), the seeding density was 22 cells/mm^2^.

Cells were fixed by adding an equal amount of 4% paraformaldehyde in PBS to the culture medium and, after 5 min, replacing the liquid with 4% paraformaldehyde in PBS for 5–10 additional minutes. After rinsing three times with PBS, coverslips were transferred onto plastic supports in a humid chamber and incubated for 15 min in blocking solution (5% normal goat serum in PBS). If permeabilization was required, the cells were post-fixed/permeabilized for 10 min in ethanol:acetic (95% absolute ethanol:5% glacial acetic acid) at −20 °C and then rinsed and blocked. For immunostaining of transmembrane proteins (such as MBP), saponin was added to the blocking solution and the primary antibody. Coverslips were incubated overnight at 4 °C with primary antibodies. After rinsing, coverslips were incubated in appropriate fluorophore-conjugated secondary antibodies (Alexa Fluor, Molecular Probes, Madrid, Spain) for 30–45 min at room temperature.

The procedure for double immunostaining depended on the antigen: when the antigen could be damaged by permeabilizing procedures (like lipidic O4 or O1 antigens), immunoreaction for these antigens was performed first, then the cells were postfixed/permeabilized in ethanol:acetic followed by immunostaining for the second antigen. When both antigens required permeabilization, antibodies were mixed in both primary and secondary reactions. Coverslips were mounted on slides with Prolong Gold (Molecular Probes, Madrid, Spain).

Monoclonal antibodies to O4 antigen were obtained as hybridoma supernatant of mouse clone O4 [29] and assayed at 1:10 dilution in blocking solution to detect membrane sulfatides in oligodendrocyte precursors. Monoclonal antibody RT97 to phosphorylated neurofilaments [30] (Developmental Studies Hybridoma Bank, maintained by the University of Iowa) were also produced as hybridoma supernatant and used at 1:10 dilution to stain axons. Anti-MBP (Myelin Basic Protein, Abcam AB7439 (Cambridge, UK) and Millipore MAB386 (Burlington, MA, USA), rat clone 12, dil. 1:100) was used to stain myelin in mature and differentiated oligodendrocytes.

### 2.7. Cell Complexity Analysis Through Fractality

To quantitatively measure cellular complexity within the oligodendrocyte lineage or to determine which pharmacological treatments enhanced oligodendrocyte differentiation, fractal analysis was applied to images of O4^+^ immunofluorescent cells in culture. This mathematical method was first utilized in 2001 to categorize the stages of oligodendrogenesis using a complexity coefficient known as the fractal dimension coefficient (D_B_) [31]. In relation to oligodendroglial differentiation, D_B_ ranges from 1.00–1.10 for OPCs to 1.50–1.70 for mature, myelinating oligodendrocytes, reflecting the increase in structural complexity.

The protocols used in the present study are detailed in Pascual-Guerra et al. [32]. In short, the fractal analysis was adapted using the box counting method, originally described by Bernard and colleagues in 2001, to fit the study’s parameters. This was performed with the FracLac Plugin (version 2.5) within the ImageJ (FIJI) software, Version 1.54, developed on the base of the study by Karperien et al. in 2013 [33] and includes a suite of image processing tools. Using a Nikon DS-FI3 camera (Nikon Instruments Inc. (Melville, NY, USA)), high-resolution pictures (2880 × 2048 px) of O4^+^ cells were randomly captured by systematic sampling (15–20 images per condition) under fluorescence microscopy with a 40× dry objective (Plan Fluorite, N.A. 0.75). After background removal and isolation of the most centrally located cell images were converted to binary (8 bits) format, and thresholding was applied to enhance the cell morphology and to eliminate background noise for the fractal analysis.

### 2.8. DRG Neurons Culture

Dorsal root ganglia (DRG) sensory neurons were obtained from E16 Sprague–Dawley rat fetuses. Given the high complexity of these dissections and cell culture procedures (including co-culture), approximately 15 different batches of DRGn were needed, just as with the iOPCs. This involved sacrificing 15 rats and dissecting the fetuses to obtain the dorsal root ganglia.

The upper body was dissected out and cleaned of viscera, exposing the ventral part of the vertebral column. Vertebral bodies were dissected and withdrawn, and the spinal cord was carefully pulled out from vertebral canal keeping the DRGs attached to the sides. DRGs were removed one by one from the cervical and upper thoracic region. The ganglia were incubated in 0.05% trypsin and 2 mM EDTA for 10–12 min and then smoothly triturated with sterile filter tips attached to a P1000 automatic pipette. Dispersed cells were obtained by passing triturated tissue through a 100 μm mesh cell strainer. For seeding, 50 μL drops of 20,000 cells/mL suspensions in Dulbecco’s minimal essential medium + 10% FBS and 50 ng/mL β-NGF (Peprotech, through ThermoFisher, Madrid, Spain) were laid at the center of poly-L-ornithine + laminin-coated round 12 mm coverslips inside 24-well plates.

Cells were allowed to attach to the coverslip surface for 15–20 min, and then medium was added to cover the well surface. Neurons spontaneously formed small clusters, leaving wide areas of the coverslip where a network of straight, nude axons grew. At 24 h, an antimitotic treatment, consisting of three 2–3 day ON and OFF cycles of 10 μM fluorodeoxyuridine and 10 μM uridine (Sigma-Aldrich) in NBB27 medium supplemented with β-NGF (50 ng/mL), was used to eliminate dividing fibroblasts, endothelial cells and Schwann cells and obtain purified neuronal cultures. After 2 weeks, these cultures contained a dense axonal framework that was used for co-culture.

### 2.9. Co-Cultures with DRG Neurons

In order to study the effects of repurposed drugs on axonal ensheathing, iOPCs and NSC- OPCs were seeded onto the neuronal cultures; these are henceforth referred to as co-cultures. Briefly, both iOPCs and OPCs were detached and disaggregated with accutase, as mentioned above, and 20,000 cells in 20 μL drops were carefully laid over neuronal cultures. In less than 10 min, all cells had adhered to the surface, most of them in contact with axons. Once seeded, the cultures were maintained for one week in NBB27 supplemented with biotin and doxycycline (in co-cultures with iOPCs) or NBB27 alone (in co-cultures with NSC-OPCs). Then, co-cultures were treated for 3 additional weeks in the same medium containing either 10 µM kainate, 1.5 µM benztropine, 1 µM miconazole, 5 µM clobetasol, 20 µM baclofen, 60 nM triiodothyronine (T_3_, positive control) or none (basal condition) in each experimental set. The culture medium with treatments was renewed every 3–4 days.

At the end of the experiment, immunocytochemistry was performed to label MBP and RT97 and obtain high-resolution photographs using confocal microscopy Nikon Eclipse Ti2 (Nikon Instruments Inc. (Melville, NY, USA)). This served to verify the wrapping of oligodendrocyte projections around neuronal axons (also confirmed with transmission electron microscopy). In this way, the effect of each drug on enhancing this ‘wrapping’ capacity was compared by counting the number and length of these myelin-wrapping tubes. To achieve this, the images were analyzed using ImageJ software (Version 1.54). The appropriate scale was applied according to the microscope’s magnification. Subsequently, the line tool was employed to obtain measurements of each enveloping tube. These tubes were then individually counted based on the analysis fields present in the confocal microscopy images.

## 3. Results

### 3.1. Kainate, Benztropine, Miconazole, Clobetasol, and Baclofen Promote the Migratory Potential of iOPCs

An essential aspect of oligodendrocyte precursor cells (OPCs) is their ability to mobilize towards injured areas to repopulate demyelinated zones. In demyelinating diseases such as multiple sclerosis, failures in the recruitment of these precursors can result in increased damage and, consequently, in the progression of demyelinating lesions.

**Figure 3:**
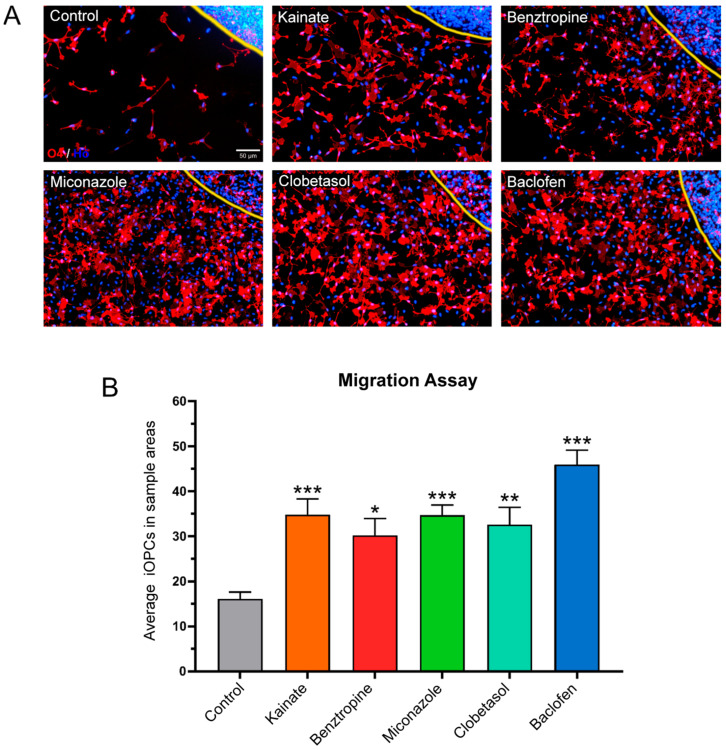
Agarose drop migration assay in iOPCs. (**A**) Representative images of O4^+^ cells migrating past the edge of the agarose drop (yellow line), under the different treatment conditions. Scale bar: 50 µm. (**B**) Number of O4^+^ cells found inside the 8 ROIs. The data represent means ± SEM (n = 20). Data were analyzed using GraphPad Prism software (Version 8.0.2) through a one-way ANOVA, applying multiple comparisons between treatment groups and the control. Specifically, the mean of each treatment group was compared to the mean of the control group. Dunnett’s test was used for post hoc correction. Asterisks indicate significant differences between the respective treatment and the control group (without repurposed drugs): * = *p* < 0.05; ** = *p* < 0.01; *** = *p* < 0.001. As shown, all five treatments provided a significant increase in iOPC migration.

Therefore, the potential impact of treatments on oligodendrocyte precursor migration was analyzed using the agarose drop seeding assay in P24 multi-well plates. Eight circular ROIs centred at 1100 µm around the edge of the seeding drop were sampled and the cell numbers in those regions were counted. This procedure provided an estimate of cell migration. It was observed that, in comparison to the control condition, all treatments facilitated a higher number of iOPCs to migrate beyond the confines of the agarose drop and reach the counting areas (Figure 3A).

**Figure 4:**
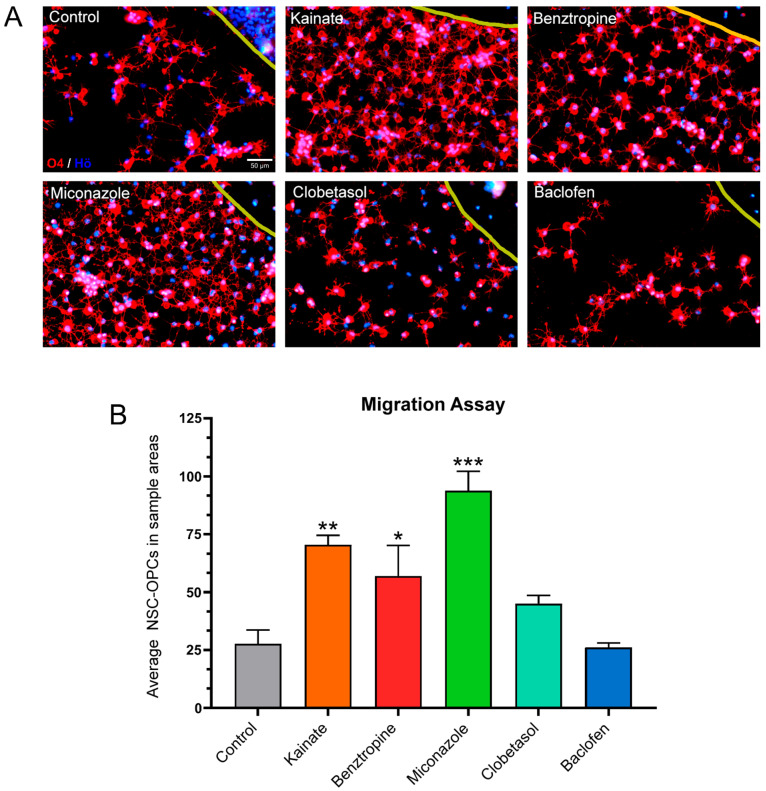
Agarose drop migration assay in NSC-OPCs. (**A**) Representative images of O4^+^ cells migrating past the edge (yellow line) of the agarose drop under the different treatment conditions. Scale bars: 50 µm. (**B**) Number of O4^+^ cells found inside the 8 ROIs. The data represent means ± SEM (n = 6). Data were analyzed using GraphPad Prism software (Version 8.0.2) through a one-way ANOVA, applying multiple comparisons between treatment groups and the control. Specifically, the mean of each treatment group was compared to the mean of the control group. Dunnett’s test was used for post hoc correction. Asterisks indicate significant differences between the respective treatment and the control group (without repurposed drugs): * = *p* < 0.05; ** = *p* < 0.01; *** = *p* < 0.001. As shown, kainate, benztropine, and miconazole significantly exhibited a higher number of migratory cells compared to the control condition.

Notably, baclofen treatment resulted in the most significant increase in the migration of O4^+^ cells (Figure 3B). When NSC-OPCs were tested under the same treatments (Figure 4A), it was observed that kainate, benztropine, and miconazole significantly stimulated the migration of O4^+^ cells. However, unlike with iOPCs, it was not observed any increase in the migration of these precursors under baclofen treatment (Figure 4B).

### 3.2. Kainate, Benztropine, Miconazole, Clobetasol, and Baclofen Enhance iOPCs Differentiation

**Figure 5:**
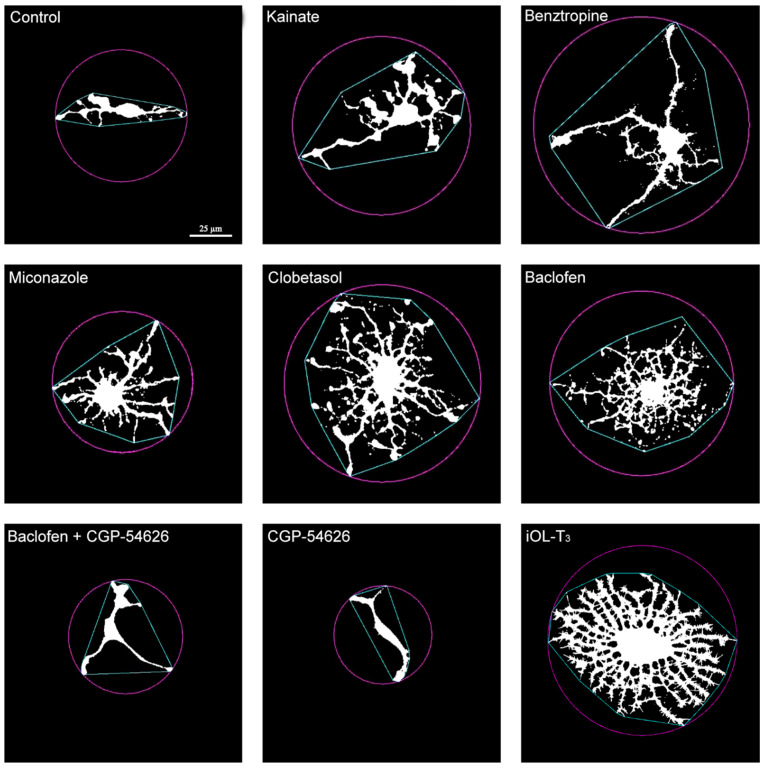
Representative images of iOPCs treated with different drugs for 7 days. The panel displays representative examples of O4^+^ cells analysed by FracLac and shows the fractal dimension coefficient (D_B_) for each cell (an average of 25 different analyses conducted per cell). All five repurposed drugs produced increased cell size and branching. Cells treated with the GABA_B_ receptor inhibitor (CGP-54626) were the smallest and less complex; in contrast, cells treated with T_3_, as a positive control producing mature iOL, exhibited the greatest size and complexity. The green lines and the purple circle that outline and encircle the cells correspond to the “convex hull and circle” of fractal analysis, respectively, indicating the minimum number of straight lines needed to enclose a cell and the smallest circular area that can encompass the cell. Scale bar, valid for all images: 25 µm.

Fractality analysis of O4 immunofluorescence, showed that iOPCs in control conditions (culture medium with growth factors) exhibited a size and morphological complexity with an average fractal dimension coefficient (D_B_) of 1.27. Under differentiation conditions (addition of 60 nM T_3_ for 7 days), iOPCs showed increased size and morphological complexity, with an average D_B_ of 1.59 (Figure 5).

**Figure 6:**
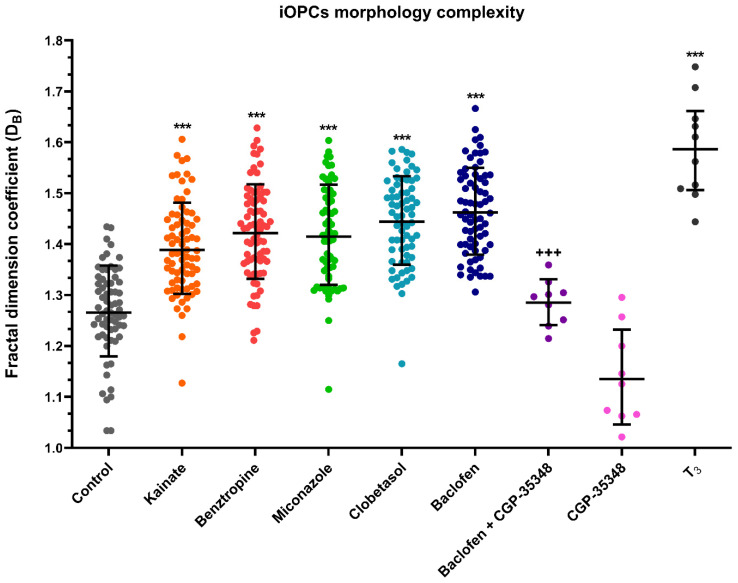
Fractal dimension coefficient data in iOPCs treated with different drugs for 7 days. Each dot corresponds to the D_B_ of an analyzed cell. Data are depicted as scatter plots for each treatment with the geometric mean ± SD. Differences between treatments were calculated using the non-parametric test of Kruskal–Wallis followed by the Dunn test. *** = *p* value < 0.001 compared to control; +++ = *p* value < 0.001 compared to baclofen. Maximum number of observations (n_max_) = 90; minimum number of observations (n_min_) = 10.

When the different tested drugs were added to the culture medium for 7 days, the cells showed a higher D_B_ in all cases. As shown in Figure 6, statistically significant differences in D_B_ compared to the control were found in all treatments, except for baclofen + CGP-54626. Additionally, significant differences in D_B_ were found between baclofen and combined baclofen + CGP-54626. Treatment with CGP-54626 alone yielded D_B_ coefficients that were smaller than the control.

**Figure 7:**
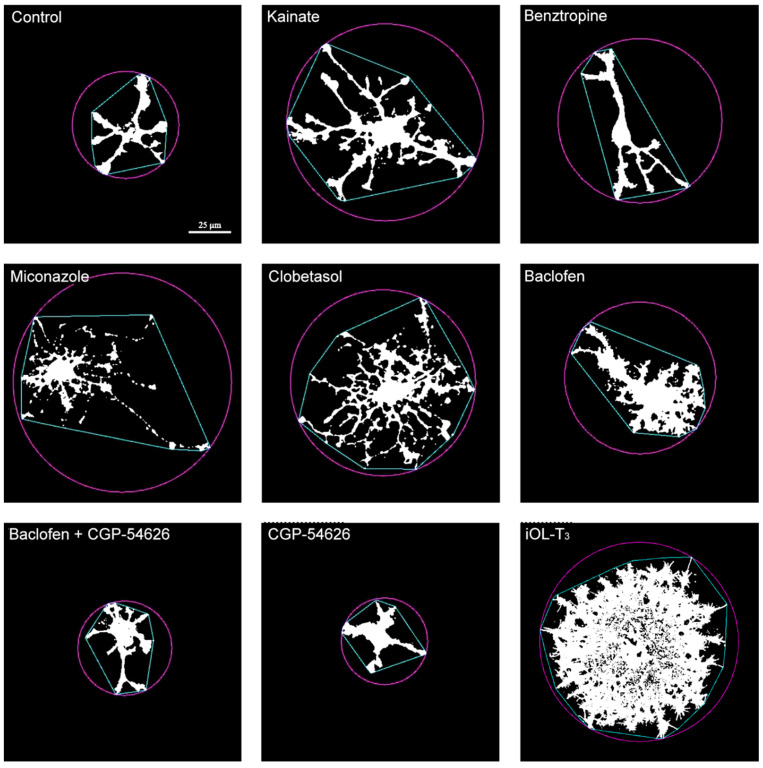
Representative images of NSC-OPCs treated with different drugs for 7 days. The panel displays representative examples of O4^+^ cells analyzed by FracLac and shows the fractal dimension coefficient (D_B_) for each cell (an average of 25 different analyses conducted per cell). In the present study, it is evident that NSC-OPCs display higher D_B_ coefficients than those of brain-extracted OPCs and of iOPCs. Cells treated with the tested drugs (benztropine being the exception) show more complex morphologies compared to the control condition. Cells treated with T_3_, as the positive control of differentiation into OL, show larger cell sizes and profuse branching in our culture conditions. Scale bar, valid for all images: 25 µm.

**Figure 8:**
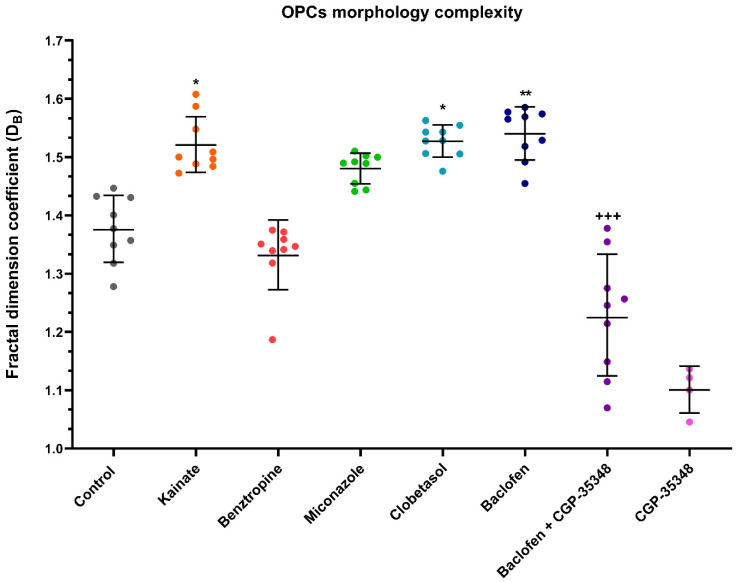
Fractal dimension coefficient data in NSC-OPCs. Each dot corresponds to the D_B_ of an analyzed cell. NSC-OPCs were cultured in NBB27 + PDGF-AA medium and treated with kainate, benztropine, miconazole, clobetasol, baclofen, baclofen + CGP-54626 or CGP-54626 for one week. Cells treated with kainate, clobetasol or baclofen showed statistically significant increases in size and complexity of morphologies in comparison to the untreated control condition. Data are depicted in a scatter plot with the geometric mean ± SD. Differences between treatments were calculated using the non-parametric test of Kruskal–Wallis followed by the Dunn test. * = *p*-value < 0.5; ** = *p* < 0.05 relative to the control condition; +++ = *p*-value < 0.001 in comparison to baclofen alone. n_max_ = 10; n_min_ = 5.

The effects of repurposed drugs on the size and complexity of NSC-OPC were also studied (Figure 7). In general, it was observed that cultured rat brain cortex-derived NSC-OPC showed a higher D_B_ than expected for OPCs [31]. This notwithstanding, our experiments showed that kainate, miconazole, clobetasol, and baclofen produced increased cell size and more complex morphologies in these NSC-OPCs (Figure 7). Additionally, as it was observed in iOPCs, when CGP-54626 was added to NSC-OPCs treated with baclofen, the D_B_ coefficient remained similar to that of control (Figure 8), while treatment with this GABA_B_ inhibitor alone produced cells with hardly any processes, yet showing no signs of cellular degeneration.

### 3.3. Kainate, Benztropine, Miconazole, Clobetasol, and Baclofen Enhance iOPCs Ensheathing Abilities

**Figure 9:**
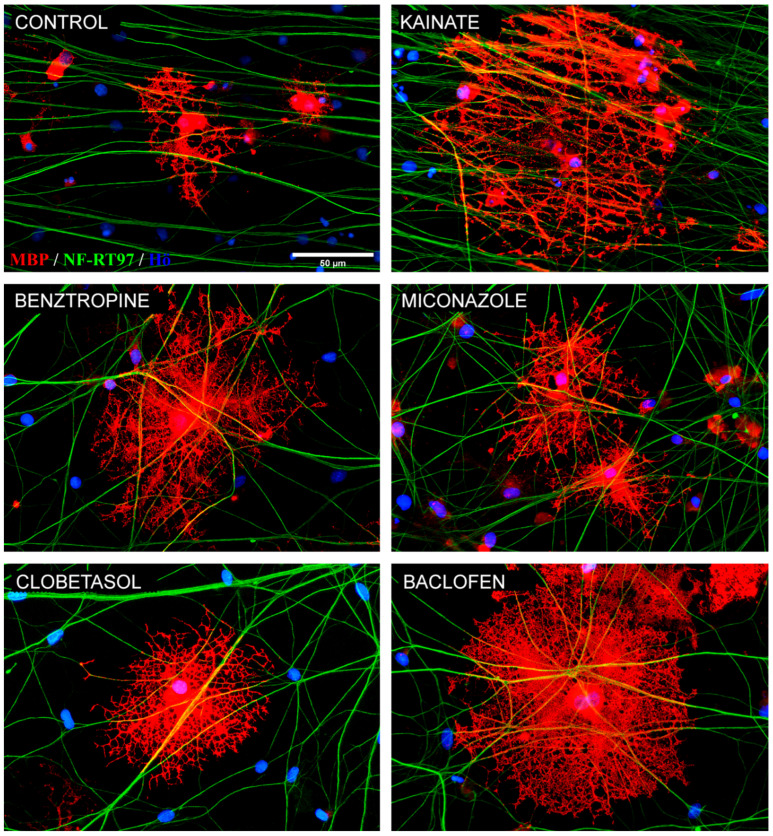
Representative images of co-cultures of iOPCs + DRG neurons for 3 weeks under various treatment conditions. Following the immunofluorescent staining for MBP (in red) and axons (neurofilament, in green), MBP^+^ cells are shown covering axonal segments of the DRG neurons. Notable differences in size, complexity, and cytoplasmic extension were observed in co-cultures treated with kainate, benztropine, miconazole, clobetasol or baclofen as compared to the control condition. In blue, cell nuclei that are stained with bis-benzimide. Scale bars: 50 µm. Note: The purple color in the images corresponds to the mixture of red (MBP) and blue (Hoechst, cell nuclei).

Myelin basic protein (MBP) and phosphorylated neurofilament (NF-RT97 clone) double immunofluorescent labeling was performed in iOPC + DRG neurons co-cultured for 3 weeks in NBB27 culture medium to quantify the pro-myelinating competence of these cells. These co-cultures showed axons being ensheathed by MBP-positive membranes. The cultures that were treated with the tested drugs generated larger and morphologically more complex MBP^+^ cells (Figure 9).

**Figure 10:**
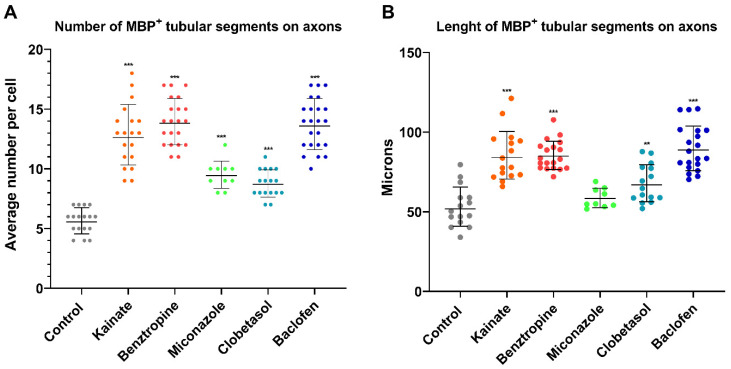
Axonal ensheathment in co-cultures under various treatments. (**A**) The number of tubular segments per MBP^+^ cell was quantified, revealing that all treatments significantly increased those numbers with respect to control. (**B**) Additionally, the length of each MBP^+^ tubular segment was increased with respect to control in all treatments, except for miconazole condition. Data are presented in a scatter plot diagram with the geometric mean ± SD. Differences between treatments were assessed by the Kruskal–Wallis test followed by the Dunn test. ** = *p* value < 0.01; *** = *p* < 0.001. n_max_ = 22; n_min_ = 10.

These differences in the treated cells were accompanied by a significantly enhanced ability to ensheath axonal segments or tracts, as evidenced by the number and length of tubular MBP^+^ segments on axons. Regarding the average number of segments ensheathed per MBP^+^ cell, all five treatments exhibited a significantly higher count than the control condition, which had an average of 5.65 segments per cell. With kainate treatment, an average of 12.84 tubular segments were counted; with benztropine, 13.95; with miconazole, 9.50; with clobetasol, 8.77; and with baclofen, 13.74 segments (Figure 10A). The average length of the tubular segments per MBP^+^ cell was also significantly increased with respect to control in all treatments, except for miconazole. As a whole, kainate, benztropine, and baclofen showed comparable pronounced increases of the number and the size of tubular MBP^+^ segments with respect to untreated cultures (Figure 10B).

**Figure 11:**
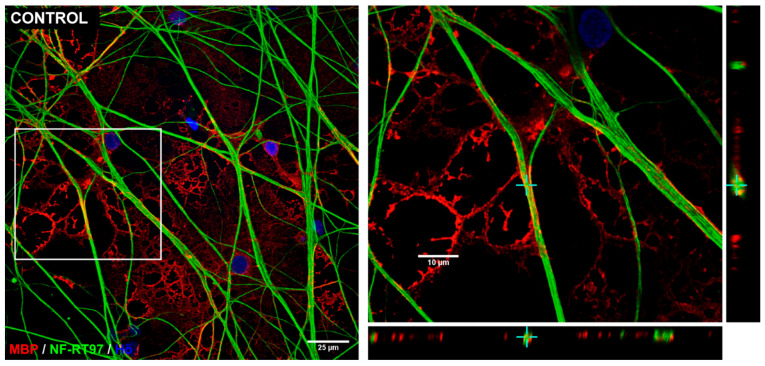

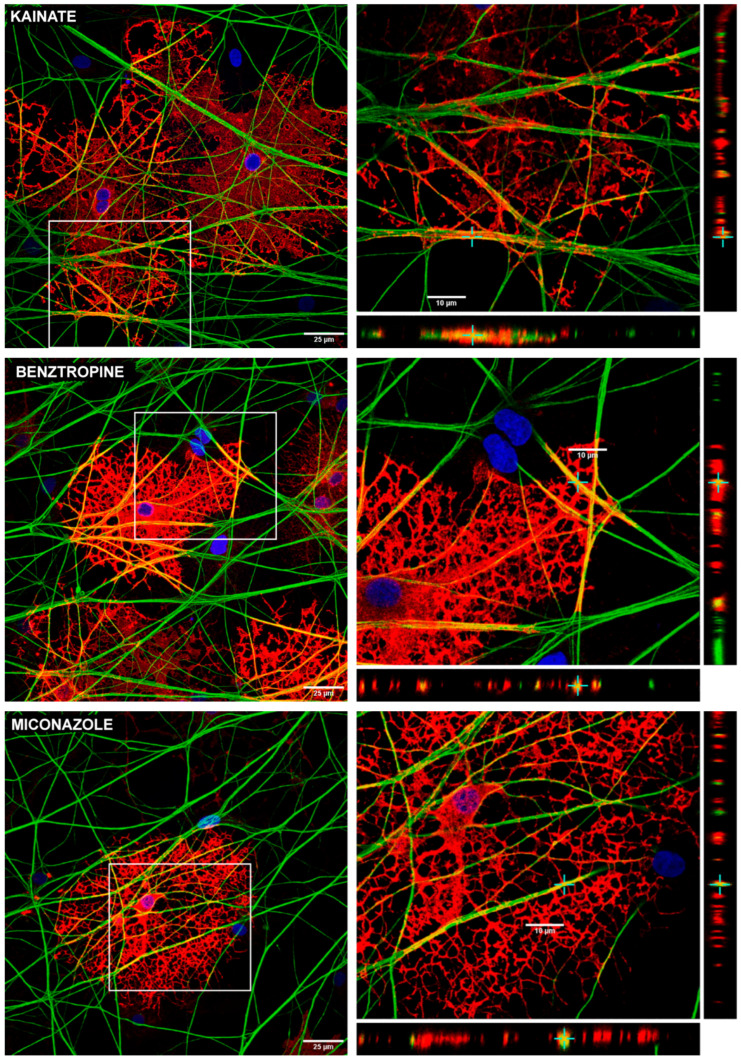

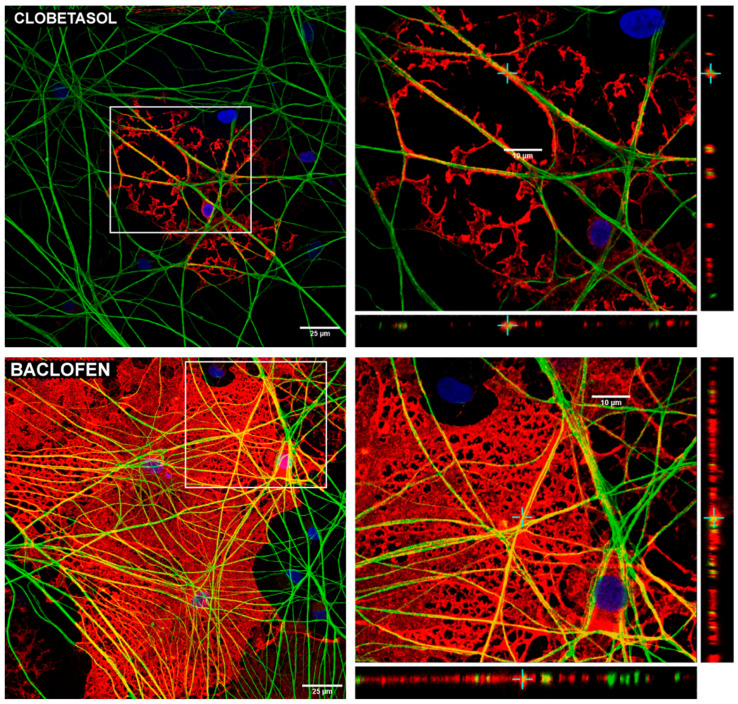
Representative images of MBP^+^ wrapping around axons. Confocal microscopy images were captured to observe the MBP immunolabeling in tubular segments around axons of DRG neurons (labeled for NF-RT97) after 3 weeks of co-culture with iOPCs. In each condition, the images on the left display the maximal fluorescence intensity projection; on the right, enlarged images of an optical section from the white box and projections in the X-Z and Y-Z axes at the level indicated by the cross to visualize the axon and its surrounding MBP^+^ sheath. Scale bars: 25 µm (images on the left); 10 µm (enlarged images on the right). Images were processed using NIS Elements software (Nikon, Melville, NY, USA), Version 6.10.01. Note: The yellow color in the images corresponds to a (co)localization of the red marker (MBP) with the green marker (neurofilament). The crosses indicate a point in the culture that was analyzed orthogonally to confirm the colocalization of both markers and, therefore, the axonal ensheathment by the induced oligodendrocyte.

To confirm the presence of axonal wrapping by these iOPCs, z-stack images were acquired using confocal microscopy. A series of images were captured at 60× magnification (N.A.: 1.40), and it was observed that, in both the control condition and the treatments with kainate, benztropine, miconazole, clobetasol, and baclofen, the axonal segments exhibited MBP^+^ profiles completely surrounding NF-RT97^+^ axons of DRG neurons (Figure 11).

## 4. Discussion

Drug repurposing is a strategy that emerges from the challenge of low productivity in the development of de novo drugs. This challenge is characterized by a significant investment of time and money that often fails to yield the anticipated returns. The underlying reasons include the low success rate of transitioning from basic and translational research to clinical application, the high costs associated with bringing a drug or therapy to market, and the extensive time required for this process. Consequently, drug repurposing offers a more expedient path to market entry. It capitalizes on the existing data from numerous databases that have already documented the pharmacokinetics, biosafety, biodistribution, and toxicity of drugs, thus bypassing many of the stages required for a newly developed drug when repurposed for a new therapeutic use [34].

In the context of demyelinating diseases, a promising therapeutic strategy involves targeting various stages of the oligodendroglial lineage. This can be achieved through cholinergic, glutamatergic, GABAergic stimuli, glucocorticoids, or the MAPK pathway, among other methods [35]. Studies have shown the efficacy of certain repurposed drugs—such as benztropine, kainate, baclofen, miconazole, and clobetasol—in promoting the differentiation and myelination of OPCs from the brains of rats or mice, or those derived from murine pluripotent cells [15,16,18,20,21,27,36,37]. However, to date, data on proliferation or migration of OPCs—critical aspects of reparative processes—remain limited.

### 4.1. Methodological Considerations

The present study makes use of several methods that we have considered appropriate to obtain a more direct measurement of the tested drugs effects on the migration, differentiation, and myelinating capability of the oligodendrocyte precursors in culture. The agarose drop method used in our study provides a quantitative estimate of cellular migratory behavior along extracellular matrices, which emulates the conditions of the pathway used by OPCs to migrate in vivo. It is an adaptation of the method proposed by ffrench-Constant’s group [28], modified to fit our experimental conditions and make it a quantitative assay.

In this method, cells are suspended at high density in melted agarose, and a small drop is laid onto a surface pre-coated with an extracellular matrix (like Geltrex or Matrigel), while keeping the agarose in a fluid condition around 37 °C. The cells sediment by gravity and attach to the extracellular matrix, confined within the agarose drop. A short cooling period then solidifies the agarose, after which the feeding medium is added to the culture well, and the plates are placed in incubators.

Migratory cells move out of the drop limits in all directions across the extracellular matrix. Cells found in eight circular areas sampled at a fixed distance from the agarose edge (Figure 2) are counted to produce data. These data are representative of the cells’ migratory activity since they are collected from areas distant from their original placement and their sampling can be considered random and systematic.

In comparison to the classical method to estimate cell migration, such as the transwell membrane crossing of the Boyden Chamber assay [38], the present method provides a more direct approach to study the capability of OPCs to move on the molecules they encounter in their CNS environment. Compared to another well-known method, the scratch assay [39], our procedure does not require a confluent cell culture and takes into account the morphological peculiarities of OPCs, among other considerations. The main limitation of this method is that it has not been widely used, so it is less known, probably because it requires some skill to set up the experiment. On the other hand, it shows experiment-to-experiment consistency in the trend of the differences between treatments and control.

Oligodendrocyte differentiation is characterized by increased membrane span, profuse branching, membrane expression of myelin proteins (such as MBP), and axonal ensheathment leading to myelination, which are distinguishing features. These characteristics can be measured in cultured oligodendroglia to assess the effects of drugs on their differentiation. Differentiation of OPCs in culture is achieved by withdrawing growth factors from the medium, and it is potentiated by the addition of T_3_. As these cells differentiate into OLs, they increase in size and display more complex morphology while retaining the O4 marker, even when they have started to express the distinctive mature oligodendrocyte membrane lipid galactocerebroside (GalC, shown by the O1 marker). Variations in cell size and branching complexity can be evaluated by calculating the cell fractality index (bidimensional in the case of cultured cells), as measured with D_B_ [31].

Here, we have used the procedure and software developed by Karperien et al. to assess the differentiation of microglia in culture [33], which was adapted to calculate the complexity of O4^+^ cells [32]. In normal oligodendroglial cultures, D_B_ may range from 1.0 (practically an unbranched sphere) to 1.8 (a large and profusely branched cell), and either parametric or non-parametric statistics within this range can be used to evaluate the level of differentiation.

The limitation of this method is that cultures need to be at low enough densities so that cells can be individualized. This may differ from the culture conditions reported in other studies, but it does not invalidate the findings.

Another limitation of our study is that the control cells were maintained in PDGF-containing medium to keep them undifferentiated. This OPC growth factor had to be added to the media receiving the tested drugs to ensure that only one variable was compared. Nonetheless, despite the cells being cultured in different media (some with PDGF without T3 and others without PDGF but with T3), the fractal analysis system reported data comparable to those of Bernard et al. [31]. As a result, the data are not distorted, nor do the DB values fall outside the expected ranges due to these differences.

Compared to the various procedures to study cellular differentiation, which are based on the surface area occupied by immunopositive cells, the D_B_ coefficient measures cell complexity individually. Therefore, cell overlapping, drug-stimulated proliferation, or cell death do not affect the measurement. Another advantage of our procedure is that O4 is the best marker to study the differentiation of OPCs to oligodendrocytes, compared to A2B5, NG2, or PDGFRα, which are down-regulated during cell maturation, or to the differentiated cell-specific markers GalC (O1) or MAG, which are not expressed in immature oligodendroglia.

The development of myelination in neuron + OPC co-cultures should ideally be assessed by electron microscopy, as it is the only method that can distinguish myelin thickness and compaction around axons. However, this approach would be impractical for screening the effects of multiple drugs due to the laborious procedures involved. Using optical microscopy (fluorescence, confocal, or even super-resolution microscopy), we can detect axonal ensheathing but not myelination. Molecular methodologies provide indirect measures but do not offer proof that all cellular processes leading to myelination are being activated. We have used the count of MBP^+^ immunofluorescent tubular profiles around DRG neuronal axons and the length of these profiles as an indirect measurement of the pro-myelinating capabilities of OPCs under different treatments.

The limitation of this procedure is that we cannot ensure that myelination will actually take place. On the other hand, the strength of this method, as shown here, is that these measurements provide an easy and consistent quantification of the tendency of OPCs to ensheath axons in culture under different drug treatments.

### 4.2. Effects of Repurposed Drugs on the Development of the Oligodendroglial Lineage of iOPCs

The present report supports that oligodendrocyte precursor-like cells, generated from adult rat ADSCs transduced to express Sox10, Olig2, and Zfp536, and here referred to as iOPCs, are responsive to the same pharmacological stimuli as CNS-derived oligodendrocyte precursors. Furthermore, it reveals that a panel of drugs previously used for other purposes, namely kainate, benztropine, miconazole, clobetasol, and baclofen, in one way or another, contributes to the migration and differentiation of these iOPCs and promotes the axonal ensheathing abilities of iOLs in vitro.

The study also corroborates that these drugs not only facilitate differentiation and myelination—as previously documented in the literature—but also enhance the migration of OPCs derived from rat NSCs.

#### 4.2.1. Kainate

Historically, kainate has been recognized as a neurotoxic agent that operates through ionotropic glutamate AMPA/Kainate receptors, leading to an increase in intracellular calcium ions akin to what is observed during CNS injury events like ischemia [40,41]. However, earlier research by Redondo and Bazán in 2007, which showed that low concentrations of kainic acid (1–10 µM) could stimulate the proliferation and differentiation of OPCs derived from rat striatal NSCs via AMPA receptors, thereby enhancing CREB phosphorylation and cAMP levels [27]. Consequently, it has been suggested that activating AMPA receptors with low concentrations of kainate may serve as a novel therapeutic avenue, in contrast to other research report advocating for the inhibition of these receptors [42].

The present study reveals that treatment with 10 µM kainate induces increased morphological complexity of O4^+^ cells, along with the promotion of both iOPC and NSC-OPC migration, and a significant improvement in axonal ensheathment (both in length and number of MBP^+^ tubular profiles in co-cultures with neurons) relative to the untreated control condition. It has been reported that, in rat brains, pre-oligodendroglial cells express a wide variety of neurotransmitter receptors, including AMPA/kainate receptors, and receive both classic and non-classic synaptic contacts with neurons that seem to regulate the activity of those cells, influencing their cell cycle, migration, differentiation, and myelination [43].

Similarly, it has been shown in vivo that white matter OPCs express glutamate receptors and receive glutamatergic signals from demyelinated axons through AMPA receptors that instruct OPCs to differentiate into new myelinating oligodendrocytes [13].

In fractal analyses, it was observed that kainate treatment increased the D_B_ coefficient in both OPCs and iOPCs compared to the control condition. Those coefficients would fit in the rank of immature-mature oligodendrocytes according to the fractal analyses performed by Bernard and colleagues in primary cultures of neonatal rat cerebellar oligodendroglia [31].

There are no previous reports showing that kainate agonism of glutamatergic receptors promotes oligodendrocyte differentiation and increases fractality. Interestingly, it has been reported that agonism of the metabotropic glutamate receptor 4 with L-AP4 promoted oligodendrocyte maturation and survival; however, in that study, kainate was probed exclusively at neurotoxic doses with no result [44].

Kainate treatment also caused the cells to migrate out of their confinement in the agarose drop through a culture surface coating of extracellular matrix (Geltrex). OPC migration has been widely described to occur during normal neurogenic development as well as after injury and demyelination events [45,46]. Migration might involve glutamatergic receptors-activated kinases linked to integrins [47] and the PLP1/α_V_-integrin complex. These mechanisms would reduce the binding of OPCs to the extracellular matrix and enhance their motility [48,49]. These studies by Gudz and collaborators also propose that myelin PLP, an OL-specific protein, is essential for neurotransmitter-induced enhancement of OPC migration, thus supporting our previous finding that iOPCs express PLP/DM20 [9].

Quantification of MBP^+^ wraps around axons in iOPC-DRG neuron co-cultures suggested that kainate also stimulated the axonal ensheathing capacities of iOPCs. As a whole, kainate at low concentrations (10 µM or less) stimulates migration, differentiation, and ensheathing in iOPCs, and may thus have the capacity to potentiate all steps leading to remyelination.

#### 4.2.2. Benztropine

In the present work, we verify that benztropine also enhances the differentiation and pro-myelinating capacities of iOPCs and, furthermore, we also show that it stimulates their migration, in the same way as it does with OPCs derived from rat cortical NSCs, which is a novelty about the effects of benztropine on oligodendroglia.

Besides its role in neurotransmission, muscarinic receptors regulate basic cellular functions such as growth, survival, differentiation, or apoptosis in different non-neuronal cells [50]. Deshmukh and collaborators proposed that benztropine pro-myelinating activity involves direct antagonism of M1/M3 muscarinic receptors, which are predominantly present in OPCs and not in mature oligodendrocytes [18]. This premise was based in the increased expression of MBP and MOG observed under benztropine.

However, the central antihistaminic action of benztropine might also explain such differentiation enhancement, given that it has been seen that histamine receptors (and more specifically H_3_) are negative regulators of the maturation and myelination of oligodendrocytes [51]. Surprisingly to us, our study was not able to confirm that benztropine enhances NSC-OPCs differentiation as no differences of their fractal D_B_ coefficients were observed when comparing to that of control, untreated cells, in contrast to what we show in iOPC cultures.

Enhanced migration was observed in benztropine-treated iOPCs and NSC-OPCs, compared to their respective untreated control cultures. Studies by other authors have observed that activity of muscarinic receptors could modulate the expression of Notch-1 [36], an important regulator of both differentiation and migration of oligodendrocyte precursors [52]. According to these views, activation of Notch-1 by muscarinic agonists would inhibit differentiation of OPCs and facilitate cell migration. Consequently, benztropine antagonism on M1 and M3 receptors should inhibit Notch-1 expression in OPCs (as shown by Deshmukh and collaborators), stimulate their differentiation, and inhibit their migration.

However, our results show that benztropine increases migration in both iOPCs and NSC-OPCs while being capable of enhancing their morphological complexity and/or the amount of axonal ensheathing. Such discrepancy would indicate that additional underlying mechanisms, probably involving PKA, Shh, MAPK or CREB may participate. Several studies show that these molecules play a relevant role in the mechanism of action of different drugs or molecules (citicoline, fingolimod, neurotrophin-3…) that promote both the proliferation and differentiation of OPCs, not necessarily being exclusive or opposed processes [53,54,55,56]. Further specific in-depth research is necessary to elucidate those mechanisms.

#### 4.2.3. Miconazole

The present study shows that miconazole enhanced the number of iOPC migratory cells, their morphological complexity, and the number of sheaths around axons (but not their length) as compared to control untreated iOPCs. It is also shown that miconazole greatly increases the number of migratory NSC-OPCs and enhances their complexity as well. These results support and extend the findings of Najm and collaborators [15] and back a possible use of miconazole as a repurposed drug to enhance remyelination.

The proposed mechanisms for these effects of miconazole are varied. Miconazole may influence migration by inhibiting the ErbB/Akt pathway, as seen in spinal cord and sciatic nerve regeneration models [57], or by increasing BDNF and CDK5 levels, which are involved in survival, migration, and neural maturation [23]. Differentiation enhancement by miconazole has been reported by a previous study, which pointed to a mechanism involving the MAPK pathway and ERK1/2 complex phosphorylation [15]. This group also studied the effect of miconazole in promoting remyelination in demyelinated animals and showed that miconazole promoted remyelination in vivo, suggesting that a physiological environment or damage stimulus might be necessary for such effects.

#### 4.2.4. Clobetasol

Using mouse epiblast stem cell-derived OPCs for screening, Najm and collaborators proposed that clobetasol, a synthetic fluorinated corticosteroid, could also be beneficial in promoting the differentiation of oligodendrocytes in vitro. When tested in autoimmune mouse models of remyelination, clobetasol demonstrated a striking reversal of disease severity, while in cultures, increased differentiation of human OPCs was observed [15]. Similar results were reported by others [15,58], which offers an alternative to its current clinical use for treating topical diseases such as psoriasis, dermatitis, or eczema. However, unlike what they observed with miconazole, those reports speculated that the remyelinating activity of clobetasol might be attributable, at least partially, to its immunosuppressant activity.

The present study aimed to verify whether clobetasol had a direct effect on migration, differentiation, and/or axonal ensheathing in iOPCs and NSC-OPCs. Clobetasol produced increased D_B_ in both iOPCs and NSC-OPCs, supporting its stimulation of oligodendrocyte precursor differentiation. This differentiation may result from glucocorticoid receptor phosphorylation at Ser220, since antagonizing this receptor with RU486 inhibits the differentiating action of clobetasol [15]. In addition to this expected differentiating activity, the effect of clobetasol on cell migration was tested. Indeed, clobetasol produced a higher number of iOPCs in migratory sampling areas, although in NSC-OPCs, we did not observe significant migration differences compared to untreated controls. These results suggest that clobetasol may stimulate cell migration besides differentiation.

In co-cultures of iOPCs and DRG neurons, clobetasol mildly but significantly increased the numbers and lengths of axonal wraps, confirming that clobetasol might favor myelination by acting on oligodendroglia, independently of its immunosuppressant activity. Supporting clobetasol’s pro-myelinating activity, it has been shown that it enhances MBP gene expression through agonism Smo and RXRγ activation [19]. However, emerging evidence suggests that, rather than acting as a conventional Smo agonist, clobetasol primarily modulates signaling through dual mechanisms: (1) inhibiting the canonical, Gli1-dependent Hedgehog pathway. Vicario et al. (2021) demonstrated that treatment with clobetasol leads to a downregulation of Gli1 expression, indicating its antagonistic effect on this pathway [59]; (2) in parallel, clobetasol activates a non-canonical signaling cascade that is dependent on AMP-activated protein kinase (AMPK) and Gli2. This alternative route results in the upregulation of genes critical for neural repair, notably RXRγ and MBP. Nocita et al. (2019) provided evidence that clobetasol enhances the expression of Gli2, RXRγ, and MBP [60]. Complementary findings by Fang et al. (2022) and Del Giovane et al. (2021) further support the concept that the non-canonical, AMPK and Gli2-dependent pathway promotes the upregulation of RXRγ and MBP, thus contributing to remyelination processes [61,62].

#### 4.2.5. Baclofen

Baclofen is an agonist of GABA_B_ receptors that has been used clinically as a muscle relaxant and antispasmodic agent. It is occasionally used to treat muscle symptoms caused by multiple sclerosis, including spasms, pain, and stiffness [63]. However, recent studies have shown that GABA_B_ receptor agonism can promote both the proliferation and migration of OPCs as well as their progress to mature myelinating oligodendrocytes [11,26,64].

In the present study, we demonstrate that baclofen acts through GABA_B_ receptors and strongly enhances the migration of iOPCs, their differentiation into mature oligodendrocytes, and their ability to wrap axons in vitro. Fractal analysis of NSC-OPCs supported that baclofen also enhances their differentiation, as previously shown by another team [65]. However, its effects on NSC-OPC motility were not demonstrated by the agarose drop migration assay, coinciding with a previous report that used CG-4 OPC cultures in an agarose drop migration assay on poly-L-lysine [11], although it did show stimulated cell migration when using fibronectin-coated transwell assays. These discrepancies do not allow us to confirm an enhancement of migration of oligodendrocyte precursors treated with baclofen, but we cannot discard it either.

Finally, baclofen-treated iOPCs co-cultures with DRG neurons showed enhanced numbers of axonal wraps, which were, on average, longer than those wrapped in untreated controls. This enhancement of myelinating capabilities (wrapping) by baclofen aligns with the studies by Serrano-Regal and collaborators, who found that this drug effectively promoted increased myelination in co-cultures of DRG neurons with OPCs derived from rat brain.

This effect appears to involve Akt phosphorylation dependent on PI3K and Src kinases [11,26,37,64]. Furthermore, baclofen has been shown to stimulate remyelination of demyelinated areas in organotypic cultures.

### 4.3. In Vitro Models to Study Pro-Myelinating Effects of Repurposed Drugs

As discussed above, drugs with very different pharmacological activities (excitatory neurotransmitter agonists or antagonists, antifungals, glucocorticoids, inhibitory neurotransmitter agonists) and intracellular mechanisms produce convergent actions on oligodendroglial cells, leading to an enhancement of their myelinating capabilities: migration, differentiation, and axonal wrapping. The experiments reported here were performed using only drug concentrations considered efficient in the literature, but different doses might have improved results.

Additionally, combinations of two or more of these drugs might further enhance their pro-myelinating actions. With that in mind, we aimed to test whether an oligodendroglial surrogate, iOPC, generated by transduction of pro-oligodendroglial transcription factors [7,66] into easy-to-produce adult rat ADSCs [9], might facilitate drug screening and dose optimization in vitro, thereby reducing the need for animal experiments.

We have previously shown that ADSC-derived iOPCs express most OPC characteristics, including myelin components, neurotransmitter receptors, metabolic responses, and axonal ensheathing capabilities [9].

In the present study, we show that these iOPCs are comparable to a large extent with brain-derived NSC-OPCs in their response to diverse repurposed drugs. However, our experiments also showed some discrepancies in those responses between iOPCs and NSC-OPCs. Such discrepancies may stem from the peculiarities of the NSC-OPC culture, such as the apparent lack of differentiation enhancement by benztropine with respect to untreated controls, likely attributable to the elevated basal fractal D_B_ of control NSC-OPCs, or the strong increment induced by baclofen on iOPC migration while showing no effect on NSC-OPC migration.

In this respect, it should be kept in mind that the heterogeneity of oligodendroglia from different CNS areas [67,68] translates into heterogeneity of experimental OPC culture models. Therefore, the in vitro preclinical assessment of the pro-myelinating effect of repurposed drugs may vary depending on the adopted OPC culture model. Our study shows that iOPC cultures may be a sensitive, experimental animal-reduced alternative for the preliminary selection of drugs and doses with pro-myelinating effects on oligodendroglial progenitors.

## 5. Conclusions

The repurposed drugs studied in the present work (kainate, benztropine, miconazole, clobetasol, and baclofen) demonstrate effects on iOPCs in terms of promoting their migration, differentiation, and ability to wrap axons in vitro to a greater extent than the untreated control condition. Considering all the necessary events for myelin reparative processes after an injury, baclofen appeared as the most effective repurposed drug in the study when tested in iOPCs. Additionally, despite functioning through different intracellular mechanisms, treatments with kainate (at low concentration) and benztropine also have notable effects on those processes. Clobetasol showed lower direct enhancing effects in vitro but might benefit from its immunosuppressant activity in vivo, and miconazole showed the highest migration fostering in NSC-OPCs. Given that these drugs act through very different intracellular mechanisms, combinations of them might produce synergistic improvement of re-myelination.

## Data Availability

The datasets underpinning the conclusions of this study are accessible through the corresponding author (JPG), although their availability may be subjected to certain restrictions. These datasets were utilized under license specifically for this investigation and are, therefore, not publicly accessible. However, the data can be obtained from the authors upon reasonable request, contingent upon approval from the Fundación Teófilo Hernando and the rest of the co-authors.

## Supplementary Materials

The following supporting information can be downloaded at: https://www.mdpi.com/article/10.3390/cells14070533/s1, Table S1: List of reactives and fungible material used in the present study.

## Abbreviations

ADSCs	Adipose-derived stromal cells
ADSC-SOZ	Sox10-Olig2-Zfp536-transduced ADSC
bFGF	Basic fibroblast growth factor
CNS	Central Nervous System
D_B_	Fractal dimension coefficient
DRGn	Dorsal root ganglia neurons
EGF	Endothelial growth factor
GABA	γ-aminobutyric acid
iOL	Induced oligodendrocytes
iOPCs	Induced oligodendrocyte precursor cells
MBP	Myelin basic protein
MS	Multiple sclerosis
NSC-OPCs	Neural stem cell-derived oligodendrocyte precursor cells
OLs	Oligodendrocytes
OPCs	Oligodendrocyte precursor cells
PDGF-AA	Platelet derived growth factor AA
